# Dietary interventions and nutritional strategies for menopausal health: a mini review

**DOI:** 10.3389/fnut.2025.1702105

**Published:** 2025-12-15

**Authors:** Yi-chen Liu, Zhi-qing Guo

**Affiliations:** 1 Xiamen Hospital of T.C.M, Xiamen, China; 2 Faculty of Health Sciences, University of Macau, Macau, China

**Keywords:** menopause, dietary patterns, Mediterranean diet, plant-based nutrition, bone health, cardiovascular risk, micronutrient supplementation, personalized nutrition

## Abstract

Menopause constitutes a pivotal physiological transition characterized by irreversible cessation of ovarian function and profound estrogen depletion, precipitating vasomotor symptoms, accelerated bone resorption, heightened cardiovascular risk, and adverse metabolic reprogramming. This mini-review systematically synthesizes evidence from 42 high-quality studies (18 systematic reviews/meta-analyses and 24 randomized controlled trials) published between January 2020 and October 2025. Studies were sourced via structured searches of PubMed/MEDLINE, Scopus, Web of Science, and Cochrane Library, focusing on dietary patterns and targeted nutrient interventions in peri- and postmenopausal women (inclusion: outcomes on vasomotor symptoms, bone health, cardiovascular risk, or metabolic parameters; exclusion: hormone replacement therapy or non-human research). Narrative synthesis incorporated critical appraisal of study quality, heterogeneity, and bias. Adherence to the Mediterranean diet consistently demonstrates clinically meaningful reductions in blood pressure and triglyceride concentrations, thereby attenuating cardiovascular burden. Calcium and vitamin D co-supplementation robustly preserves bone mineral density and mitigates fracture risk. Plant-based dietary patterns rich in whole grains, fruits, and vegetables are associated with attenuated symptom severity and enhanced healthy aging trajectories. Despite these advances, postmenopausal women remain disproportionately vulnerable to micronutrient deficiencies, underscoring the imperative for balanced, nutrient-dense intake. These non-pharmacological strategies confer substantial improvements in quality of life, yet persistent gaps in long-term outcomes and representation of diverse populations necessitate further investigation.

## 1 Introduction

Menopause represents a critical physiological milestone in female aging, typically occurring between 45 and 55 years, defined by the permanent cessation of menstruation consequent upon ovarian follicular exhaustion and marked estrogen decline (1). This endocrine transition precipitates a cascade of multisystem disturbances, encompassing vasomotor instability (hot flashes and night sweats), accelerated bone mineral loss with heightened osteoporosis risk, adverse lipid remodeling, endothelial dysfunction, increased visceral adiposity, insulin resistance, and dyslipidemia (2). These manifestations profoundly impair health-related quality of life and substantially elevate the lifelong burden of cardiometabolic and skeletal disease, projected to affect 1.2 billion women globally by 2030 (3).

Nutrition has emerged as a cornerstone of menopausal health management, offering evidence-based, non-pharmacological modalities capable of modulating hormonal, inflammatory, and metabolic pathways (1). Diets enriched with anti-inflammatory and phytoestrogen-containing foods have been repeatedly linked to amelioration of vasomotor symptom severity and preservation of bone integrity (4). Moreover, optimal nutrient intake counteracts sarcopenia, supports cognitive resilience, and promotes healthy longevity (5). Yet, postmenopausal women exhibit heightened susceptibility to deficiencies in calcium, vitamin D, magnesium, iron, and antioxidant micronutrients—deficiencies exacerbated by diminished dietary intake, impaired absorption, and sedentary lifestyles (6). Such inadequacies are particularly pronounced in low- and middle-income settings, perpetuating global health inequities (7).

## 2 Methodology

This mini-review adopted a structured, narrative synthesis approach targeting high-quality evidence published between January 2020 and October 2025. PubMed/MEDLINE, Scopus, Web of Science, and Cochrane Library were systematically searched using predefined terms. Inclusion criteria comprised: (1) systematic reviews/meta-analyses or RCTs; (2) primary focus on dietary patterns or specific nutrient interventions in peri- or postmenopausal women; (3) outcomes encompassing vasomotor symptoms, bone health, cardiovascular risk factors, or metabolic parameters; (4) English language publication. Exclusion criteria included studies centered on hormone replacement therapy, non-human research, or grey literature. After deduplication and dual title/abstract screening, 123 full texts were evaluated, yielding 42 studies (18 systematic reviews/meta-analyses and 24 RCTs) for final inclusion. Evidence was narratively synthesized with critical appraisal of study quality, (using AMSTAR-2 for reviews and RoB 2 for RCTs), heterogeneity (e.g., *I*^2^ statistics where reported), and risk of bias. Subgroup analyses from included studies were considered for diverse populations where reported.

The primary objectives were threefold: (1) to consolidate contemporary evidence on efficacious dietary patterns and nutrient strategies; (2) to critically examine controversies surrounding food-derived versus supplemental sources; and (3) to delineate persistent research gaps, including longitudinal outcomes, underrepresentation of non-Western populations, and the imperative for genomically informed personalized nutrition.

### 2.1 Nutritional needs and challenges in menopausal women

Estrogen depletion triggers profound physiological reprogramming that fundamentally alters nutritional requirements and metabolic homeostasis (8). Postmenopausal women typically experience accelerated weight gain—predominantly central adiposity—driven by a declining resting metabolic rate, unfavourable redistribution of fat, and progressive loss of lean muscle mass, collectively amplifying the risk of insulin resistance and metabolic syndrome (9). Concurrently, bone mineral density undergoes rapid decline, markedly increasing osteoporosis susceptibility, while systemic low-grade inflammation intensifies, manifesting as joint pain, heightened cardiovascular strain, and an overall escalation of symptom burden (8, 10). These alterations are compounded by age-related impairments in gastrointestinal absorptive capacity and lifestyle-related reductions in physical activity, which collectively exacerbate nutritional vulnerability (9).

Micronutrient deficiencies are highly prevalent in this population, with postmenopausal women at substantially elevated risk of inadequate intake or absorption of calcium, vitamin D, magnesium, iron, and antioxidant compounds, frequently attributable to suboptimal dietary patterns and disrupted nutrient metabolism secondary to hormonal changes (11, 12). Vitamin D deficiency, in particular, is ubiquitous, arising from limited sun exposure, insufficient dietary sources, and impaired renal 1α-hydroxylation, thereby disrupting calcium homeostasis and compromising skeletal integrity (13). Although menstrual iron losses cease, deficiency remains common due to low dietary intake or malabsorption, contributing to fatigue and anaemia; magnesium inadequacy, likewise, is implicated in muscle cramps, sleep disturbance, and mood dysregulation (8, 10, 14, 15). Epidemiological data reveal these deficiencies are especially pronounced in low- and middle-income settings and among ethnically diverse populations with limited access to nutrient-dense foods, thereby perpetuating global health disparities (9).

Evidence-based guidelines therefore advocate targeted increases in specific macro- and micronutrients. Protein requirements rise to 1.1–1.5 g/kg body weight daily to preserve lean mass and support metabolic function, preferably from high-quality sources such as lean meats, legumes, and dairy (16, 17). Calcium intake should achieve ≥1,200 mg/day, combined with vitamin D 800–2,000 IU/day, to attenuate bone loss, with supplementation indicated when dietary sources prove insufficient (18). Contemporary dietary challenges include low fibre intake (associated with worsened vasomotor symptoms) and excess saturated fat (which promotes inflammation and adiposity) (19, 20). Overcoming these barriers demands culturally sensitive, individualised nutrition education that prioritises integral whole-food matrices, yet socioeconomic, educational, and cultural constraints continue to impede optimal adherence (10).

Table 1 summarises the key nutrients, evidence-based intake recommendations, primary dietary sources, and principal benefits for postmenopausal women. These recommendations align with 2025 guidelines from NIH ODS, NAMS, EFSA, and WHO guidelines.

**Table 1:** Recommended daily intakes, food sources, and benefits of key nutrients for menopausal women.

Nutrient	Recommended daily intake^*^	Food sources	Benefits	Ref.
Calcium	1,200 mg	Dairy products, leafy greens, fortified plant milks	Supports bone density, reduces osteoporosis risk	(18)
Vitamin D	800–2,000 IU	Fatty fish, eggs, fortified foods, sunlight exposure	Enhances calcium absorption, prevents bone loss	(13)
Protein	1.1–1.5 g/kg body weight	Lean meats, beans, nuts, dairy	Maintains muscle mass, aids weight management	(16)
Iron	8–18 mg (if deficient)	Red meat, lentils, spinach	Prevents anemia, combats fatigue	(10, 14, 15, 18)
Magnesium	320 mg	Nuts, seeds, whole grains	Reduces inflammation, improves sleep quality	(72, 73)

*Recommendations based on 2025 NIH ODS, NAMS, EFSA, and WHO guidelines.

### 2.2 Dietary patterns and their impact on menopausal symptoms

Dietary patterns exert profound influence on the menopausal transition by modulating hormonal milieu, systemic inflammation, and metabolic homeostasis (10). Contemporary evidence from systematic reviews and high-quality randomized controlled trials consistently demonstrates that sustained adherence to specific dietary frameworks can meaningfully attenuate vasomotor symptoms (including hot flashes and night sweats), ameliorate mood disturbances, facilitate weight regulation, and substantially mitigate risk of chronic diseases such as cardiovascular disease and osteoporosis (21). These patterns uniformly prioritise whole foods, dietary fibre, and potent anti-inflammatory constituents, thereby providing accessible, non-pharmacological interventions optimally suited to the physiological demands of postmenopausal women (8).

Among these, the Mediterranean diet emerges as the most robustly evidenced paradigm, distinguished by abundant intake of fruits, vegetables, whole grains, legumes, nuts, extra-virgin olive oil, and moderate consumption of fish and dairy products. Meta-analytic synthesis of interventions in menopausal cohorts reveals strong associations with diminished overall symptom severity, preserved skeletal integrity through enhanced bone mineral density, and favourable cardiovascular remodelling manifested by reductions in blood pressure and triglyceride concentrations (22). Higher adherence scores, in particular, correlate with marked attenuation of vasomotor symptom intensity and improved lipid metabolism—effects plausibly mediated by the diet’s high polyphenol and long-chain omega-3 fatty acid content, which collectively suppress oxidative stress and inflammatory signalling (23). Longitudinal observational data further substantiate the Mediterranean diet’s capacity to foster healthy ageing by counteracting the metabolic dysregulation characteristic of the postmenopausal state (24).

Plant-predominant dietary patterns, which emphasise fruits, vegetables, whole grains, and legumes while minimising animal-derived foods, similarly demonstrate considerable promise in alleviating both depressive symptomatology and vasomotor disturbances (25, 26). Narrative and systematic reviews attribute these benefits to abundant phytoestrogens and soluble fibre, which stabilise glycaemic excursions and modulate endogenous hormone fluctuations (21). Low-fat, high-fibre regimens—exemplified by the Women’s Health Initiative Dietary Modification Trial involving over 17,000 postmenopausal participants—likewise confer modest yet clinically relevant reductions in vasomotor symptoms through enhanced intake of whole grains, fruits, and vegetables coupled with restriction of saturated fats, thereby supporting weight homeostasis and metabolic health (27). The Dietary Approaches to Stop Hypertension (DASH) pattern, sharing substantial overlap with the Mediterranean diet yet placing additional emphasis on sodium restriction and potassium-rich foods, exhibits comparable efficacy in mitigating the accentuated cardiovascular risk profile of menopause.

Notwithstanding these salutary effects, important controversies persist regarding cross-cultural adaptability and long-term sustainability. Although the Mediterranean diet demonstrates unequivocal efficacy in Western populations of European descent, its translation to Asian cohorts is frequently constrained by entrenched culinary traditions and consequently diminished adherence, necessitating culturally congruent modifications (23). Moreover, the preponderance of extant evidence derives from observational cohorts, with relatively sparse high-quality randomized trials in ethnically diverse or socioeconomically disadvantaged groups, thereby limiting generalisability and underscoring the urgent need for more inclusive, long-duration interventional research (28).

### 2.3 Key nutrients and supplementation strategies

Menopause markedly heightens susceptibility to micronutrient deficiencies, thereby necessitating strategic, targeted intake to preserve skeletal integrity, cardiovascular function, and metabolic homeostasis (9). Contemporary systematic reviews strongly advocate prioritising food-derived nutrients while employing supplementation judiciously to rectify deficiencies, particularly in populations with habitually suboptimal dietary patterns (29). This paradigm effectively circumvents the hazards of over-supplementation, including potential adverse cardiovascular sequelae and hypercalcaemia.

Calcium and vitamin D constitute the foundational pillars for osteoporosis prevention in postmenopausal women, in whom estrogen withdrawal dramatically accelerates bone resorption (30). Co-supplementation reliably augments bone mineral density and attenuates fracture risk, with evidence-based recommendations specifying ≥1,200 mg calcium and 800–2,000 IU vitamin D daily (31–34). A comprehensive 2025 systematic review confirms that titrated regimens of vitamin D and calcium yield clinically meaningful benefits in postmenopausal osteoporosis management, albeit with efficacy modulated by baseline deficiency status and adherence (30). Food-first approaches—emphasising dairy products, fortified plant-based milks, and leafy green vegetables—are unequivocally preferred, as they minimise the gastrointestinal intolerance frequently associated with high-dose supplements (35).

Anti-inflammatory micronutrients, notably omega-3 fatty acids, vitamin K (particularly menaquinone-7), selenium, and magnesium, exert critical roles in counteracting the heightened inflammatory and cardiovascular burden of menopause (9). Omega-3 supplementation, especially eicosapentaenoic acid-rich formulations, consistently lowers triglyceride concentrations and systemic inflammatory markers, conferring robust cardioprotection (36). Long-term menaquinone-7 administration has been shown to ameliorate arterial stiffness and hypertension (37), while selenium and magnesium bolster antioxidant defences and sleep architecture, with the latter additionally alleviating muscle cramps and anxiety symptomatology (10, 38, 39). Optimal sources include fatty fish (omega-3), natto and green leafy vegetables (vitamin K), Brazil nuts (selenium), and whole grains/seeds (magnesium).

Additional compounds such as iron and *β*-carotene address persistent anaemia risk and oxidative stress, respectively (40–42). Although menstrual iron losses cease, deficiency remains prevalent due to inadequate dietary intake and age-related absorption decline, warranting vigilant monitoring to avert fatigue and cognitive sequelae (43). Elevated *β*-carotene intake, through abundant fruit and vegetable consumption, is associated with reduced depressive symptoms and hypertension in menopausal cohorts (44, 45).

Controversies persist regarding the relative merits of supplementation versus dietary sources. Whereas vitamin D supplementation is unequivocally efficacious and safe when appropriately dosed, demonstrating consistent gains in bone mineral density and fracture risk reduction without substantive adverse effects, excess supplemental iron in women aged >50 years may precipitate oxidative stress, chronic inflammation, and accelerated cellular ageing pathways (46–48). This risk arises from diminished post-menopausal iron requirements coupled with poorer absorption and bioavailability of inorganic supplemental forms, which bypass physiological hepcidin regulation and predispose to tissue accumulation. In contrast, non-heme iron from integral whole-food matrices is subject to tight homeostatic control, substantially mitigating overload risk and reinforcing the primacy of dietary approaches (49). Broader concerns surround the heterogeneous quality and regulatory oversight of dietary supplements, with documented cases of hepatotoxicity and contaminant exposure further underscoring the preference for nutrient-dense whole foods as the sustainable cornerstone of sustainable menopausal nutrition (50). Important research lacunae remain, particularly regarding long-term cognitive implications of omega-3 and iron status, where preliminary signals of benefit await confirmation from adequately powered trials in ageing female populations (49, 51).

### 2.4 Evidence summary and intervention recommendations

In synthesising the extant evidence, dietary patterns and targeted nutrient intake emerge as robust, empirically substantiated paradigms for attenuating menopausal symptomatology and forestalling attendant comorbidities. Sustained adherence to Mediterranean and plant-predominant diets consistently yields clinically meaningful reductions in vasomotor disturbances, cardiovascular jeopardy, and skeletal demineralisation, mediated by synergistic anti-inflammatory and antioxidant cascades. Pivotal micronutrients—calcium, vitamin D, and omega-3 polyunsaturated fatty acids—further amplify these salutary effects, with judicious supplementation demonstrating unequivocal efficacy in rectifying deficiencies where dietary provenance proves inadequate. Nonetheless, the preponderance of observational designs underscores the imperative for expanded randomised controlled trials to delineate causality and affirm protracted therapeutic durability.

Intervention imperatives centre on bespoke nutritional architectures, seamlessly integrating dietary frameworks with adjunctive lifestyle modalities—such as structured aerobic and resistance exercise—to optimise metabolic homeostasis and symptom palliation. Exemplifying this, the synergistic coupling of Mediterranean dietary fidelity with resistance training robustly safeguards lean mass, bolsters bone mineral density, and facilitates weight homeostasis. Clinicians are enjoined to interrogate individual micronutrient deficits through biomarker profiling and calibrate counsel to sociocultural milieus, privileging integrous whole-food matrices over synthetic supplements to circumscribe iatrogenic hazards. Prospective paradigms may harness digital phenotyping and adherence-tracking algorithms to cultivate enduring behavioural congruence, thereby engendering enduring enhancements in health-related quality of life among menopausal cohorts.

## 3 Discussion

This mini-review synthesises contemporary evidence, illuminating nutrition’s indispensable role in modulating menopausal health trajectories by deftly alleviating symptomatology and forestalling chronic comorbidities. Sustained fidelity to Mediterranean and plant-predominant dietary paradigms consistently engenders clinically salient reductions in vasomotor perturbations, cardiovascular vulnerability, and osseous demineralisation, underpinned by synergistic anti-inflammatory and metabolic modulatory cascades (8, 21, 27, 52). Indispensable micronutrients—calcium, vitamin D, and omega-3 polyunsaturated fatty acids—further augment these salubrious effects, with integrated interventions demonstrably augmenting bone mineral density while curtailing fracture propensity (8). In toto, equilibrated nutrition not only circumvents acute exigencies such as thermoregulatory instability and adiposity accrual but also cultivates enduring enhancements in quality of life and longevity, engendering resilience against insidious comorbidities including osteoporosis and atherosclerotic sequelae (53–56).

Debates endure concerning the relative merits of food-derived versus supplemental nutrient provenance, with empirical signals intimating that integrous whole-food matrices confer safer pharmacokinetic profiles—particularly for iron, via hepcidin-mediated absorptive regulation—while supplements retain utility in rectifying profound deficits (57). Dietary modalities proffer holistic dividends, albeit with efficacy modulated by individual comportment and baseline physiological milieu (58). Methodological limitations pervade the corpus, evinced by overreliance on observational architectures that preclude causal attribution, alongside paucity of protracted randomised controlled trials to assay sustained therapeutic fidelity (59, 60). Compounding this, representational lacunae abound, with preponderant data emanating from Western demographies that elide non-Western women, whose symptom constellations may diverge by virtue of genetic, sociocultural, and ecophenotypic idiosyncrasies (61).

The preponderance of extant evidence remains Western-centric, constraining its translatability to non-Caucasian cohorts wherein symptom phenomenology diverges markedly: African American women, for instance, report more frequent and severe vasomotor symptoms than their Caucasian counterparts, potentially deriving amplified benefit from phytoestrogen-replete paradigms akin to soy-centric Asian dietary motifs (62). Regionally, Mediterranean archetypes necessitate bespoke localisation—e.g., supplanting extra-virgin olive oil with cost-efficacious canola or mustard variants in South Asian milieus—to augment palatability and feasibility (63). Socioeconomic strictures, encompassing food insecurity afflicting approximately 30% of postmenopausal women in low- and middle-income countries, straitjacket procurement of nutrient-dense comestibles, thereby widening health schisms; decentralised programmatic scaffolds, such as subsidised plant-based staples and community kitchens, proffer pragmatic palliatives (64). Imperative prospective inquiries must stratify by ethnicity and socioeconomic stratum to unmask these pivotal moderators, thereby engendering equitable, culturally congruent interventions (65).

Prospective hurdles to nutritional operationalisation encompass suboptimal adherence precipitated by entrenched lifestyle impediments, culinary dissonances with prescriptive paradigms (e.g., Mediterranean tenets in Asian milieus), and socioeconomic strictures curtailing procurement of nutrient-replete victuals in resource-constrained locales (8). Cross-cultural variegations in help-seeking for menopausal malaise—often encumbered by stigma or informational asymmetries—further attenuate uptake of empirically validated stratagems (61). Remediation demands polyvalent architectures: tailored nutraceutical counselling and behavioural priming to bolster compliance, ethnoculturally attuned dietary scaffolds to augment pertinence, and decentralised programmatic initiatives to redress access asymmetries (8). Synergising nutrition with ambulatory modalities, such as resistance and aerobic exercise, amplifies dividends, as corroborated in perimenopausal wellness consortia (66, 67).

Gazing forward, prospective vectors must exalt precision and bespoke nutrigenomics, harnessing genomic, metabolomic, and artificial intelligence-augmented analytics to calibrate interventions consonant with individual endocrine, physiological, and alimentary idiosyncrasies (68). Ubiquitous wearables and telemetric platforms hold promise for real-time symptom surveillance and adherence orchestration, facilitating iterative refinements for superlative outcomes (69, 70). Amplifying randomised controlled trials to encompass marginalised cohorts, while probing nexuses with neurocognitive and cardiometabolic domains, will redress pivotal interstices (71). Clinicians, in turn, are exhorted to champion Mediterranean-inflected dietary archetypes alongside comprehensive nutraceutical pedagogy, thereby empowering women to traverse menopause with agency and equity (8). In bridging these interstices, nutritional stratagems possess the transformative potential to reconfigure menopausal stewardship as a personalised, resilient paradigm.
